# TBP-related factors: a paradigm of diversity in transcription initiation

**DOI:** 10.1186/2045-3701-1-23

**Published:** 2011-06-27

**Authors:** Waseem Akhtar, Gert Jan C Veenstra

**Affiliations:** 1 Radboud University Nijmegen, Department of Molecular Biology, Faculty of Science, Nijmegen Centre for Molecular Life Sciences, Nijmegen, The Netherlands

## Abstract

TATA binding protein (TBP) is a key component of the eukaryotic transcription initiation machinery. It functions in several complexes involved in core promoter recognition and assembly of the pre-initiation complex. Through gene duplication eukaryotes have expanded their repertoire of TATA binding proteins, leading to a variable composition of the transcription machinery. In vertebrates this repertoire consists of TBP, TBP-like factor (TLF, also known as TBPL1, TRF2) and TBP2 (also known as TBPL2, TRF3). All three factors are essential, with TLF and TBP2 playing important roles in development and differentiation, in particular gametogenesis and early embryonic development, whereas TBP dominates somatic cell transcription. TBP-related factors may compete for promoters when co-expressed, but also show preferential interactions with subsets of promoters. Initiation factor switching occurs on account of differential expression of these proteins in gametes, embryos and somatic cells. Paralogs of TFIIA and TAF subunits account for additional variation in the transcription initiation complex. This variation in core promoter recognition accommodates the expanded regulatory capacity and specificity required for germ cells and embryonic development in higher eukaryotes.

## Introduction

Eukaryotic cells have three RNA polymerases: RNA polymerase I (RNAP-I) transcribes the ribosomal RNA genes, RNAP-II mostly transcribes protein-coding genes and some non-coding genes, whereas RNAP-III generally transcribes small RNA genes. The RNA polymerases, however, do not have the ability to recognize the proper sites of transcription initiation. For the recruitment of each RNA polymerase to the site of initiation, an additional set of polypeptides is required, the basal transcription factors. These proteins recognize the core promoter, a sequence of approximately 100 bp around the transcription start site that is required for initiation of transcription and which contains specific sequence motifs, such as the TATA box, Initiator (Inr) and Downstream Promoter Element (DPE) [1]. The basal transcription factors for RNA polymerase II (RNAP-II) include Transcription Factor II A (TFIIA), TFIIB, TFIID, TFIIE, TFIIF and TFIIH (reviewed in [2]) which together with RNAP-II form the pre initiation complex (PIC). PIC formation typically starts with the recognition of the core promoter by TFIID, which consists of TATA box binding protein (TBP) and up to 14 other polypeptides called TBP-associated factors (TAFs) [2]. TFIID and other basal transcription factors have been considered a general set of proteins involved in transcription at all RNAP-II promoters. However, TFIID is present in many forms in different tissues and cell types. These forms have different variants of TAFs as well as distinct additional polypeptides ([3] and references therein). Similarly, in many species TBP is not the universal initiation factor that it is in yeast. Repeated gene duplication events have produced TBP-related factors in different species. In this essay we discuss a new paradigm of versatility of the transcription machinery, which involves selective factor recruitment as well as factor switching in the context of the expanded regulatory complexity of germ cell and embryonic development and differentiation.

## TATA box binding protein (TBP)

**Figure 1:**
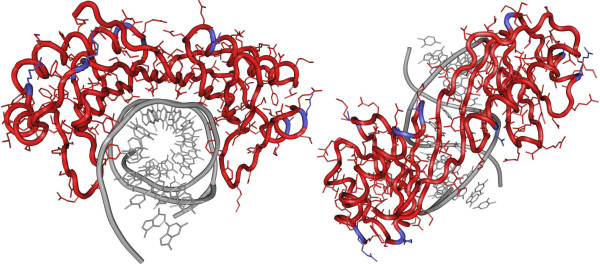
**Structure of core domain of human TBP bound to the TATA element of the adenovirus major late promoter**. The DNA is shown in grey, TBP is shown in red. Amino acids that are different between TBP and TBP2 are shown in blue. Left: Frontal view, showing that all amino acids contacting the DNA are identical between TBP and TBP2 (red). Substituted amino acids are solvent exposed (blue). Right: Top view, showing that TBP aligns with the minor groove in which it is inserted.

**Figure 2:**
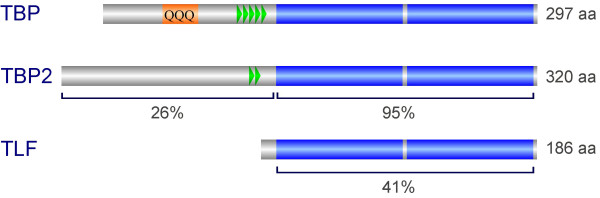
**Domain structure of the vertebrate TBP family**. The core domain (blue) is formed by an imperfect direct repeat (blue) and is involved in binding to the TATA box in the case of TBP and TBP2. TLF lacks an N-terminal domain. The N-terminal domain of TBP2 is not as well conserved as the core domain. The PXT repeats in the N-terminal domain are indicated (green), as well as the poly-glutamine stretch of TBP (orange).

**Figure 3:**
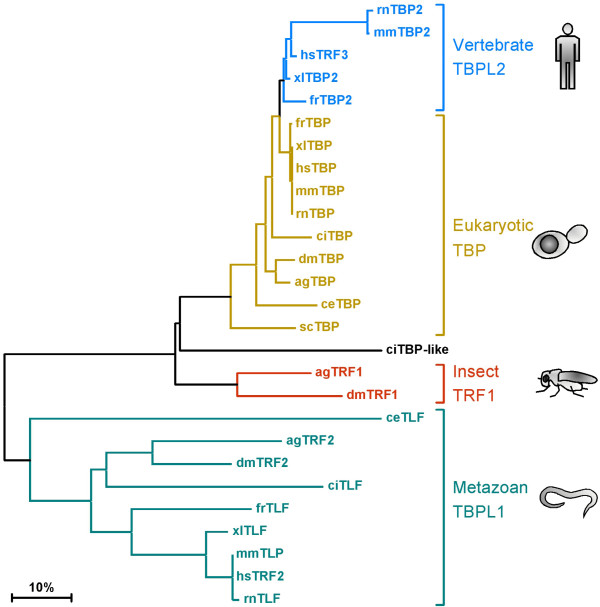
**Phylogenetic tree of the TBP-related factors in eukaryotes based on alignment of their core domains**. TBP (gold) is the founding member of the family with representatives in archae (not shown), yeast and all metazoans. TRF (TRF1, red) is found in the fruit fly and the mosquito. TLF (TBPL1, TRF2, light green) is found in all multicellular organisms, but not in yeast. TBP2 (TBPL2, TRF3, blue) is restricted to vertebrates. The bar signifies the distance in the tree corresponding to 10% divergence. Legend: hs-*Homo sapiens*, mm-*Mus musculus*, rn-*Rattus norvegicus*, xl-*Xenopus laevis*, fr-*Fugu rubripes*, ci-*Ciona intestinalis*, dm-*Drosophila melanogaster*, ag-*Anopheles gambiae*, ce-*Caenorhabditis elegans*, sc-*Saccharomyces cerevisiae*. ciTBP: XP_002127120, ciTLF: XP_002128047, ciTBP-like: BW175023, agTBP: XP_309748, agTRF1: XP_317373, agTRF2: XP_308361.

TBP is the founding member of the family and is involved in transcription by all of the three major RNA polymerases (I, II and III) in eukaryotes [4]. It is conserved from archea to man. The primary structure of TBP consists of two major domains. The core domain, which is highly conserved (80% identical between yeast and mammals) consists of two direct pseudo repeats and folds into a saddle-shaped structure with a convex and a concave surface (Figures 1, 2). The convex surface of this saddle binds to the minor groove of the TATA box via an induced fit model [5]. The N-terminal domain is relatively variable in sequence and size between species but is conserved in vertebrates. TBP is the founding member of small family of TBP paralogs in eukaryotic genomes (Figure 2, 3).

### Structure of TBP

High resolution crystal structures of the core domain of TBP from different species have been solved [6-12]. These structural studies illustrate that the core domain folds into a bipartite saddle like structure with approximate dimensions of 32 × 45 × 60 angstroms (Figure 1). The saddle consists of two quasi-symmetric halves each consisting of a five-stranded, anti-parallel curved β sheet and two α helices that lie perpendicular to each other. Four β strands from each half of the saddle form the concave underside that binds to DNA. The four α helices, two from each half of the saddle, the basic peptide that links the two quasi-symmetric domains and parts of two β strands make up the convex upper side of the saddle. This surface harbors the binding interfaces for many proteins involved in the process of transcription initiation.

### TATA box recognition by TBP

TBP binds to the TATA box via an induced-fit mechanism of protein-DNA recognition [8-10,12,13]. The DNA is highly bent and TBP also goes through a conformational change. The DNA enters the C-terminal portion of TBP as a regular B-DNA conformation (corresponding to the 5'-end of DNA) but then undergoes a transition into a unique partially unwounded right-handed double helix by a kink. This kink in DNA is caused by the insertion of two phenylalanine residues (Phe284/Phe301 in human TBP) into the minor groove between the first two base pairs of the TATA box. A similar kink is produced at the exit of the DNA from the other side by the insertion of two other phenylalanine residues (Phe193/Phe210) between the 7^th ^and the 8^th ^base pairs of the TATA box. The second kink restores the DNA back to its usual B-DNA conformation. In this way, while bound to TBP, the middle six base pairs of the TATA box are in a partially unwound right-handed double helical conformation with a widened minor groove which fits in an energetically favored manner to the concave surface. TBP binding to a canonical TATA box proceeds via a two-step pathway; first an unstable complex with unbent DNA is established. Second this complex is slowly converted into a stable complex in which the DNA is bent [14]. The second step is greatly accelerated by TFIIB in cooperation with an inhibitory DNA binding surface of TBP.

### TBP complexes

TBP is part of a number of complexes which function with different RNA polymerases. To function at RNAP-I promoters, TBP joins the Selectivity factor 1 (SL1) complex together with five RNAP-I specific TAFs (reviewed in [15]). TBP and two RNAP-III-specific TAFs (BrfI and BdpI) assemble into the TFIIIB complex, which is involved in RNAP-III transcription. In vertebrates, there are two versions of TFIIIB complex containing either BrfI or its homolog BrfII (reviewed in [16]). Besides TFIIIB, TBP also interacts with the snRNA activating protein complex (SNAPc) at the human U6 promoter [17]. At many RNAP-II promoters TBP works in a large complex called TFIID, which contains in addition to TBP approximately 14 evolutionarily conserved distinct RNAP-II specific TAFs (reviewed in [18,19]). The TFIID complex recognizes the RNAP-II core promoter as a result of recognition of different core promoter elements by TBP and TAFs and starts the nucleation of the PIC. Additionally, it also serves as a targeting interface for the binding of many transcription activators. However, the activity of TBP at RNAP-II promoters is also modulated through the formation of alternative complexes as discussed in the next paragraph.

### Negative modulation of TBP activity

TATA boxes and AT-rich sequences are scattered over the genome and TBP has a relatively low specificity for binding to the TATA box. The affinity for its DNA element is ~1000 times lower than the proteins that bind the major groove of DNA [20]. In addition, TBP has a very low dissociation rate after it has bound to DNA. This implies that there should be mechanisms in place to negatively modulate the ability of TBP to bind DNA as well as to actively remove TBP from its DNA-bound state.

Two distinct domains in the N-terminal region of TAF1, the largest subunit of TFIID, interact with TBP and negatively modulate its ability to bind TATA box [21]. TAF N-terminal domain 1 (TAND1) binds to the concave surface of TBP and thus directly blocks DNA binding [22]. Additionally, the TAND1 binding surface also overlaps with the binding site of transcriptional activator VP16 resulting in a competition between the two proteins to bind TBP [23]. TAND2 binds to an interface on the convex surface of TBP that is crucial for TFIIA binding. Thus, TAND2 negatively influences the binding of TBP with TFIIA [24], which otherwise would stabilize binding of TBP to the TATA box.

TBP also forms a distinct complex involved in RNAP-II transcription called B-TFIID, in which TBP interacts with a unique TAF called BTAF1 (reviewed in [25]). It is called Mot1 in yeast and is a Swi2/Snf2-related ATPase that exerts both positive and negative effects on transcription [26-28]. Very much like TAF1, it also binds to the concave and convex surface of TBP and thus modulates TBP binding to DNA and TFIIA respectively [29]. An important function of BTAF1 is to dissociate the TBP-DNA complex using the energy of ATP hydrolysis [30]. In this way BTAF1 can also clear TBP from spurious TATA boxes and redistribute it to bona fide promoters, which partially accounts for BTAF1's ability to positively affect transcription at some promoters [27,28]. Another well-known TBP complex is formed by its association with negative cofactor 2 (NC2). NC2 consists of two subunits called NC2α (Dr1) and NC2ß (Drap1) which are bound with each other via histone-fold domains [31]. NC2 binds to free TBP as well as TBP bound to DNA. The NC2-TBP-DNA ternary complex locks the TBP in a state that is resistant to binding with TFIIA and TFIIB [32]. NC2 and Mot1 were shown to inhibit transcription from TATA-containing promoters but stimulated the transcription from DPE-dependent promoters [33,34]. Interestingly, the presence of a functional initiator sequence can provide resistance against NC2-mediated transcriptional repression [35]. A genome wide occupancy analysis in yeast has shown that TBP, NC2, and Mot1 colocalize at transcriptionally active RNAP-II core promoters. ATP hydrolysis by Mot1 disassembles this complex resulting in dissociation of TBP from DNA suggesting that NC2 and Mot1 cooperate to control the dynamics of TBP turnover *in vivo *[36]. Interestingly, this turnover of TBP at TATA box-containing promoters (often associated with tightly regulated genes) is faster in comparison to TATA-less promoters, implicating TBP removal in stringent regulation of transcription [37,38].

### Role of N-terminal domain of TBP

TBPs from vertebrates contain a conserved N-terminal domain (Figure 2). *In vitro *studies have shown that this domain is important for TATA-containing RNAP-II and III promoters but dispensable for TATA-less transcription [39]. Furthermore, this domain negatively modulates the binding of the TATA box by the core domain and mediates cooperative binding with SNAPc at the U6 promoter [14,40]. Contrary to these *in vitro *findings, homozygous loss of most of the N-terminal domain has no apparent effect on either cell growth or transcription by RNAP-II and RNAP-III [41]. However, the N-terminal domain plays an important role in post-transplantation embryos to evade rejection by the maternal immune response by repressing a β2 microglobin mediated process [42]. The mechanism underlying this phenomenon remains to be elucidated. The N-terminal domain is also clinically important as it features a glutamine repeat region (Figure 2), the expansion of which results in spino-cerebellar ataxia 17 (SCA17, reviewed in [43]).

### TBP function through the cell cycle

Changes in TBP levels affect the transcription of genes involved in the cell cycle. TBP expression is often elevated in Ras oncogene-transformed cells. Concordantly, stable over-expression of TBP leads to transformation of cells [44,45]. Inversely, reduction of TBP levels to 50% by inactivating one of its alleles in chicken DT40 lymphoid cells results in a decrease in the expression of cdc25B phosphatase. This leads to an increase in hyperphosphorylated Cdc2 resulting in delayed cell cycle and enhanced apoptosis [46]. In addition, TBP plays an important role during mitosis in the transmission of active gene memory to daughter cells [47,48]. The underlying mechanism involves TBP-mediated recruitment of protein phosphatase 2A, which locally inactivates condensin via dephosphorylation and thus inhibits compaction of transcriptionally active gene loci during mitosis [48].

### Requirement of TBP in early development

**Figure 4:**
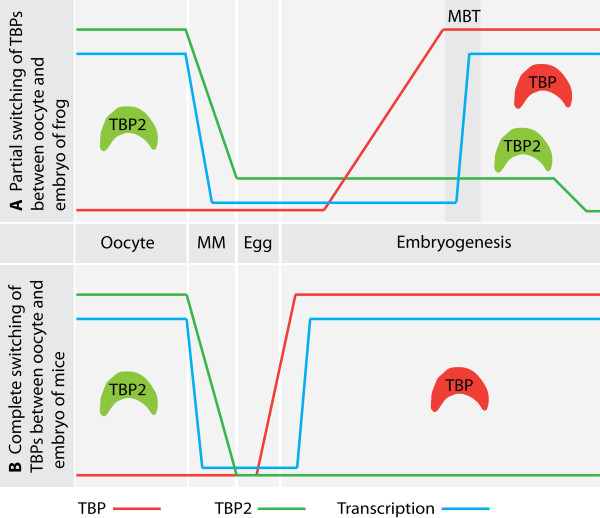
**Switching of TATA binding proteins between oocytes and embryos**. (A) In *Xenopus *oocytes TBP2 but not TBP is present at the protein level. Upon meiotic maturation (MM) TBP2 is degraded after global shut down of transcription, but residual levels of TBP2 persist in egg and early embryo. During early cleavages after fertilization, maternal stores of TBP mRNA are translated and by the mid-blastula transition (MBT) both TBP and residual TBP2 contribute to zygotic transcription leading to a partial switching of function between TBP and TBP2. In *X. laevis *TBP2 RNA is detected well into larval stages, whereas in *X. tropicalis *TBP2 mRNA levels decline during gastrulation (our unpublished data). (B) Mouse oocytes also express only TBP2. When zygotic transcription commences at the two cell stage of the mouse embryo, TBP2 function is replaced by translationally upregulated TBP leading to a complete switching of TATA binding factors.

TBP mRNA is abundant in oocytes, but the encoded protein is not. The TBP messenger is translationally masked in oocytes and is only translated during early cleavage stages [49]. TBP protein levels peak at the *Xenopus *mid-blastula transition, at the onset of zygotic transcription. Likewise, TBP protein only accumulates in 2-cell mouse zygote, at the onset of mouse zygotic gene activation [50,51] (Figure 4). Precocious translation of recombinant TBP mRNA allows pre-MBT promoter activity in frog embryos, but only in combination with competitor DNA, which competes for chromatin assembly [49,52].

Not all transcription in early embryos (zebrafish, *Xenopus*, mouse) requires the presence of TBP [53-55]. Even though TBP is essential, it is necessary for transcription of only a small subset of genes in *Xenopus *embryos [56]. Also in mouse zygotes, not all transcription requires TBP [53]. The other two vertebrate TBP-related factors (TBP2 and TLF) are not thought to be involved [53,57,58] so the underlying mechanism of TBP-independent transcription in mouse zygotes remains to be solved.

Some of the embryonic TBP-dependent genes regulate degradation of maternal mRNAs through the miR-430 pathway in zebrafish [59]. The fact that TBP is not expressed in the oocyte but is important for both the onset of new transcription at the mid-blastula transition and the degradation of maternal messages, implies that this factor plays a crucial role in the transition from maternal to zygotic control of gene expression [59]. Many of the transcripts that depend on TBP in the embryo are also expressed maternally and in adult stages, have orthologs in yeast, and show no functional specialization [56], suggesting that the founding member of the animal TBP family is a relatively generic initiation factor that is required for both embryonic and somatic cell transcription [56,59].

## TBP-related factor

TBP-related factor (TRF, also known as TRF1) was the first TBP paralog to be discovered in animals [60]. TRF1 was discovered in a genetic screen of *Drosophila *and turns out to be specific to insects (Figure 3). It is highly expressed in embryonic central nervous system and male germ cells [61]. It is 63% identical to TBP in the core domain, can associate with the TATA box and stimulate basal transcription *in vitro *[62], whereas *in vivo *it can direct transcription from one of the *tudor *gene promoters [61]. Most of TRF1 is associated with the *Drosophila *homolog of the RNAP-III specific transcription factor Brf1 [63] and TRF1-depleted *Drosophila *Schneider line-2 cell extracts are unable to support RNAP-III transcription. These observations were further confirmed by genome-wide chromatin immunoprecipitation (ChIP) analysis, which shows that TRF1 is recruited to a majority of RNAP-III and some RNAP-II promoters in the fly genome [64]. Thus, in *Drosophila *cells, TFIIIB activity (an essential ingredient of RNAP-III mediated transcription) is accommodated by Brf1 and TRF1, which is in clear contrast to the situation in yeast and human cells where Brf1 is in a complex with TBP [65].

## TBP-like factor

TBP-like factor (TLF, also known as TBPL1 and TRF2) is a more distant paralog of TBP that is found in all metazoans (Figure 3). It has only ~40% identity with the TBP core domain [66] (Figure 2). It is the only member of TBP family which lacks the ability to bind the TATA box. It however, interacts with TFIIA and TFIIB [67-69]. Compared to TBP, TLF is less conserved between distinct species. The core domains of TLF are only 40-45% identical in metazoans, whereas the TBP core domain is 80% identical from yeast to human [70]. TLF forms a complex with TFIIA in HeLa cells but can also interact with TFIIA-like factor (ALF), which is a germ cell-specific paralog of TFIIA [69,71,72]. TLF was also shown to negatively modulate transcription by sequestering TFIIA [73-75]. Surprisingly, the interaction of TLF with TFIIA activates the transcription of the TATA-less *neurofibromatosis type 1 *gene [73]. On a similar note, chicken TLF was shown to inhibit transcription from TATA-containing promoters but activate transcription from TATA-less promoters [74].

The function of TLF has been studied in worm, frog and fish using knockdown and dominant negative strategies [54,55,70,76]. In these organisms, ablation of TLF function results in an early arrest of embryonic development and down regulation of a subset of genes. TLF knockdown combined with transcriptome profiling in frog embryos showed that a large number of transcripts require TLF but not TBP or the vertebrate-specific paralog TBP2 [56]. A significant proportion of these genes is preferentially expressed in embryos and shows a functional specialization in catabolism. A function in embryonic catabolism may be especially relevant in the light of the fact that TLF is important for externally developing embryos (fly, fish, frog) whereas TLF is dispensable for normal embryogenesis in mouse [58,77]. It is however, required for proper spermatogenesis as male TLF-null mice are sterile. This may suggest a functional specialization of TLF in different species. In TLF knockout mice spermatogenesis is arrested when round spermatids mature into elongated spermatids. TLF is required for proper chromocenter formation in haploid round spermatids [78]. Several other lines of evidence also link TLF to germ cell differentiation and proper chromatin formation. In *Drosophila *embryos TLF has been reported to be a part of a large protein complex containing components of nucleosome remodeling factor (NURF) complex and the fly-specific DNA replication element binding factor (DREF) [79]. TLF promotes the transcription from an upstream promoter of proliferating cell nuclear antigen (PCNA). This promoter has a DRE motif, which is bound by DREF. Intriguingly, the DRE is among the most prevalent core promoter elements in the *Drosophila *genome [80,81]. Two different isoforms of TRF2 were identified in *Drosophila*, however no differences in their expression pattern were observed indicating that they perform similar functions [72]. Partial inactivation of TLF in flies leads to defects in embryonic development, impaired premeiotic chromosome condensation and defective differentiation of male and female germ cells [72,82]. Furthermore, TLF depletion in *Drosophila *salivary glands causes severe disruption of chromatin structure in polytene chromosomes, again suggestive of a link between TLF expression and proper chromatin function [83]. In *Drosophila *TLF controls the transcription from one of the histone H1 genes, whereas the other histone genes in the cluster are TBP-dependent [83]. A genome-wide TLF occupancy analysis in *Drosophila *S2 cells revealed that TLF is bound to more than a thousand sites [83]. About 80% of these sites are not overlapping with TBP binding regions suggesting that a fair number of genes in *Drosophila *are controlled independently of TBP. Interestingly, TLF binding sites are enriched for DREs and are mostly lacking TATA boxes [83].

Taken together, TLF most likely provides an alternative mechanism of transcription initiation. The details of this alternative mechanism of initiation are not yet known but may involve chromatin remodeling. A requirement for TLF in germ cells is conserved from fly to mouse, whereas TLF is also essential for early embryogenesis in all non-mammalian metazoans examined (worm, fly, fish, frog) but not in mouse.

## TBP2

TBP2 (also known as TBPL2, TRF3) is the most recently identified member of TBP family. It has only been found in vertebrate genomes (Figure 3) and is the most closely related TBP paralog showing 95% sequence identity in the core domain (Figures 1, 2) [84,85]. It can bind to the TATA box, interacts with TFIIA and TFIIB and promotes basal transcription *in vitro *[84,85]. Although some early reports suggested a widespread expression of TBP2, it has become apparent that the main site of TBP2 expression is in oocytes, with lower but detectable expression of TBP2 RNA and protein in early embryos and embryonic stem cells, a pattern conserved in zebrafish, frog and mouse [50,84-88]. The absence of TBP protein in oocytes and the abundance of TBP2 in this cell type, suggested that TBP2 functions as a TBP replacement factor in oocytes [85]. TBP2 binds to the transcriptionally active loops of the meiosis I lampbrush chromosomes of *Xenopus *oocytes [86]. TBP2 promotes RNAP-II transcription in oocytes, but ectopically expressed TBP can do the same, showing that switching of initiation factors can be driven by expression levels in this system. Most of the oocyte TBP2 protein is actively degraded during meiotic maturation of oocytes, however, some residual maternal TBP2 remains in early embryos [85,86] (Figure 4). The first functional studies in fish and frog showed that TBP2 is indispensable for embryonic development [84,85]. A requirement for TBP2 is found particularly among vertebrate-specific genes expressed during embryogenesis, implicating this initiation factor in developmental innovation in vertebrates; TBP2 is involved in ventral specification during frog development [56]. A function for TBP2 has also been reported in differentiation pathways in zebrafish and mouse [89,90]. During zebrafish embryogenesis, TBP2 binds and promotes transcription of the *mespa *gene, which encodes a transcription factor that initiates a pathway required for the commitment of ventral mesoderm to the hematopoietic lineage [90]. This function of TBP2 is dependent on its interaction with TAF3 [91]. In contrast, TBP2 knockout mice are perfectly viable with no overt defects in embryogenesis or somatic cell differentiation. Rather the loss of TBP2 severely affects oocyte folliculogenesis rendering female *Tbp2^-/- ^*mice sterile [57]. So in mouse the most physiologically relevant function of TBP2 is in oocytes where it is most abundant, whereas in frog and fish TBP2 is also essential for embryogenesis.

## TBP family members: specialized or redundant?

The presence of functional TBP paralogs in metazoans raises the question why more than one TATA binding factor is required in these organisms while only one such protein is sufficient for proper execution of the transcriptional program in yeast. One plausible hypothesis, supported by global gene expression analysis, is the requirement of additional components of the basal transcription machinery in order to accommodate a more elaborate gene regulatory framework in these organisms that is linked to development in metazoans [56]. To accommodate specific developmental roles, one would think that each of these proteins is involved in transcription of specific subset of genes. However, the relationship between these initiation factors and regulated genes, as observed in early *Xenopus *embryos, is highly complex. On one hand TBP, TBP2 and TLF are all required for proper development and essential for specific subsets of genes [55,56,85]. On the other hand TBP-ablated embryos can be at least partially rescued by overexpression of TBP2 suggesting a significant level of redundancy between the two proteins [85].

Additionally it has also been observed that different promoters of the same gene are controlled by different TBP family members. An example is the *tudor *gene of *Drosophila*, one promoter of which is TBP-responsive whereas the other is controlled by TRF1 [62]. Similarly, TLF/TRF2 and TBP work on two different promoters of the *PCNA *gene [79]. In addition to these specific examples, other data is also suggestive of more than one TBP family member working on the same gene. In ChIP-chip assays in *Drosophila *S2 cells, it was observed that 20% of TLF binding sites overlap with TBP binding sites [83]. Furthermore, on many genes in early frog embryos both TBP and TBP2 can be located on same genes by similar assays [56]. These examples highlight the regulatory complexity in higher organisms.

## Switching of TBP family members during development and differentiation

Another intriguing explanation for the existence of multiple TATA binding factors in metazoans is spatio-temporal control of gene expression where different factors control gene expression in different tissue types or in different stages of development [92,93]. The idea is that when cells differentiate, the expression levels of one TATA binding factor decrease whereas those of another increase, effectively switching the gene-regulatory control from one factor to the other. A prime example of such a switch is the replacement of TBP by TBP2 in female germ cells (Figure 4). In frog and mouse oocytes TBP2 is abundant with little if any TBP present [50,86]. As early embryogenesis proceeds TBP levels increase reaching to their maximum at the time of zygotic gene activation [49,50]. TBP2 is actively degraded to low levels during meiotic maturation of oocytes [86]. In frogs the switching is not complete as low levels of TBP2 persist for some time and are essential for embryogenesis [56,85]. Interestingly, when TBP is ectopically expressed in oocytes, it can promote transcription [86], showing that expression differences can drive initiation factor switching. This is in contrast to another subset of specific promoters where the requirements of the specific TATA-related proteins are non-redundant even when co-expressed, as is the case with strictly TBP2-specific promoters in early frog embryos [56]. It should be noted that early mouse embryos do not tolerate the overexpression of TBP2 [57], whereas in *Xenopus *embryos, which in contrast to mouse embryos require TBP2, this factor can partially rescue a knockdown of TBP. Moreover overexpression of TBP2 in the presence of normal levels of TBP does not interfere with normal development in this species [85], suggesting a higher plasticity in core factor usage in *Xenopus *as compared to mouse.

In another but more controversial example, the canonical TFIID complex is replaced by a smaller 150 to 200 kDa complex consisting of TBP2/TRF3 and TBP-associated factor 3 (TAF3) when mouse myoblasts differentiate into myotubes in culture [89]. However, muscle differentiation does not require TBP2 *in vivo *[57] and TBP2 expression is not detected outside germ cells and early embryos by other laboratories. Also, TAF3 is a histone fold (HFD)-containing TAF and it is surprising that in the TRF3-TAF3 complex no HFD-containing partner-TAFs have been identified. Using *in vitro *re-constituted transcription systems, it was shown that a recombinant TBP2-TAF3 complex could promote transcription from the *Myogenin *promoter, whereas a TFIID complex substoichiometric for TAF3 could not [94], highlighting a role for TAF3 in MyoD-dependent transcriptional activation of *Myogenin*.

## TBP-related factors and germ cell transcription

There is increasing evidence that exquisitely specialized mechanisms of gene expression operate in germ cells. These germ cell-specific programs of transcription are facilitated not only by specialized isoforms of sequence-specific transcription factors but also by variation in the core transcription machinery. As reviewed in previous sections female germ cells lack detectable levels of TBP protein with abundant TBP2, which is essential for their development. Parallel to TBP2 in oocytes, TLF is essential for sperm development. TBP2 knockout male mice and TLF-lacking female mice are fertile showing strict division of labor between the two factors in mammals. In lower vertebrates, on the contrary, both TLF and TBP2 are also important for proper embryogenesis as detailed above.

**Figure 5:**
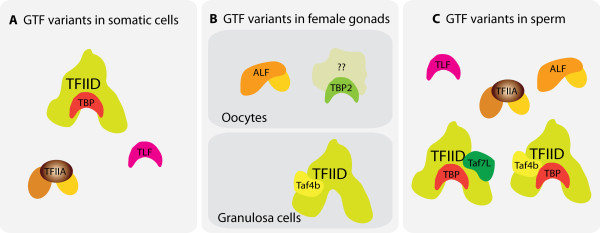
**Many germ cell-specific variants of general transcription factors (GTFs) have evolved**. Only factors are shown for which a role has been documented. While TBP dominates transcription in somatic cells (A) and is also expressed in testis (C), it is completely replaced by TBP2 in female germ cells (B). Male germ cells express high levels of TLF and a vital share of transcription in these cells is dependent on this factor. Similarly TFIIA is replaced completely in oocytes and partially in sperm by ALF. Variants of TFIID contain germ cell-specific TAFs.

This germ cell-restricted expression pattern of TBP-related factors is also accompanied by variations in the composition and expression of some other key components of core transcription machinery (Figure 5). For example TFIIA-like factor (ALF) is an exclusively germ cell-specific isoform of core transcription factor TFIIA [95]. In fact in frog oocytes ALF completely replaces TFIIA, a situation that parallels replacement of TBP by TBP2 [96]. A number of TBP-associated factors have germ cell-specific isoforms. TAF4b, a paralog of TAF4, is highly expressed in mouse testis and ovary. It is part of an ovary-specific form of TFIID which is specifically found in the granulosa cells but not developing oocytes [97]. Deletion of TAF4b in knockout mice results in completely sterile females, and males which become sterile very early in their life [97-99]. TAF7L is highly expressed in spermatogonia. However, it remains in the cytoplasm and only translocates to the nucleus in pachytene cells and haploid spermatids before becoming silenced in post-meiotic spermatocytes [100]. Similarly sperm-specific isoforms of TAF4, 5, 6, 8 and 12 have been reported in *Drosophila *[101,102].

Presence of highly specialized versions of core transcription machinery components in germ cells raises this fundamental question why these cells need a different core transcription apparatus. One possibility is that meiosis requires a specialized environment for transcription. Meiotic cell division is unique to germ cells. The expression of both TBP2 and TLF is highest and that of TBP is the lowest during the meiotic phases of germ cell development. For instance TLF protein is detectable during early pachytene stages of spermatocytes where it is principally cytoplasmic [78]. Later it migrates to the nucleus and stays there until the shutdown of transcription in elongated spermatids. Similarly, TBP2 is also abundant during transcriptionally active phases of meiosis in oocytes [50,57,86].

TBP-related factors may support chromatin remodeling by controlling the expression of key chromatin regulators at important time points during the gametogenesis. A lack of TLF results in defective chromocentre formation [78], whereas the absence of TBP2 in oocytes leads to chromatin disorganization and defects in condensation [57]. Ovaries of TBP2 knockout mice exhibit down regulation of genes involved in chromatin organization including oocyte-specific linker histone H1, H1foo, and the DNA methyltransferase Dnmt1 [57]. Confocal analysis shows that TLF is associated with both heterochromatin and euchromatin [78]. These results illustrate the importance of TLF and TBP2 for chromatin in germ cells.

## Perspective: The TBP family as a paradigm in diversity of the transcription machinery

The transcription initiation machinery in not universal but variable in composition in higher eukaryotes, which is mainly on account of duplicated paralog subunits of the canonical TFIID and TFIIA complexes. The TBP family presents a prime and well-characterized example of how non-universal mechanisms of transcription initiation contribute to cell-specific developmental gene expression programs. This diversification most likely serves to expand the gene regulatory potential required for embryonic development in metazoans (TLF), and vertebrates in particular (TBP2). Initiation factor switching occurs on account of differential expression of these proteins in gametes (TBP2 and TLF), embryos (TBP, TBP2 and TLF) and somatic cells (transcription dominated by TBP). Mice however have lost the genetic requirement for TLF and TBP2 during embryogenesis. An important goal of future research is to decipher the molecular mechanisms underlying the specificity of factor-promoter interaction, both at the level of core promoter recognition and at the level of protein-protein interactions. TBP is known to work in several multi-subunit complexes. For other members of the family this area has not been fully explored. Germ cell and embryonic gene regulation involve gene regulatory networks that integrate intercellular signaling and epigenetic regulation to reprogram chromatin state and produce activators and repressors required for pluripotency and induction of lineage commitment. The transcription initiation machinery is adapted to mediate the versatility required for the transcription program of germ cell and embryonic development. Elucidation of the molecular mechanisms underlying the diverse biological roles of the TBP family factors, using classical biochemistry, molecular biology and state-of-the-art genomics approaches, will provide deeper insight in the role of transcriptional regulation in pluripotency and differentiation in higher eukaryotes.

## List of abbreviations

ALF: TFIIA-like factor; DPE: downstream promoter element; DRE: DNA replication element; DREF: DRE-binding factor; Inr: initiator element; NURF: nucleosome remodeling factor; PCNA: proliferating cell nuclear antigen; PIC: pre-initiation complex; SL1: selectivity factor 1; SNAPc: snRNA activating protein complex; TAF: TBP-associated factor; TBP: TATA box binding protein; TFIIA: transcription factor II A (RNAP-II initiation factor); TFIIB: transcription factor II B (RNAP-II initiation factor); TFIIIB: transcription factor III B (RNAP-III initiation factor); TFIID: transcription factor II D (RNAP-II initiation factor); TLF: TBP-like factor; MBT: mid-blastula transition; TRF: TBP-related factor; RNAP: RNA polymerase.

## Competing interests

The authors declare that they have no competing interests.

## Authors' contributions

WA wrote the manuscript and made figures. GJCV contributed to the writing and made figures. Both authors read and approved the final manuscript.
